# *Ex vivo* propofol permeation across nasal mucosa: A proof-of-concept study for outpatient light sedation via nasal route

**DOI:** 10.5599/admet.2403

**Published:** 2024-08-16

**Authors:** Michele D. Spampinato, Anna Costanzini, Roberto De Giorgio, Angelina Passaro, Nicola Realdon, Fabrizio Bortolotti, Sabrina Banella, Gaia Colombo

**Affiliations:** 1 Department of Translational Medicine, St. Anna University Hospital, University of Ferrara, 44124 Ferrara, Italy; 2 Department of Pharmaceutical and Pharmacological Sciences, University of Padova, 35131 Padova, Italy; 3 Department of Life Sciences and Biotechnology, University of Ferrara, Via Fossato di Mortara 19, 44121 Ferrara, Italy

**Keywords:** Sedative drug, nasal administration, aqueous solubility, n-cyclodextrin, transmucosal permeation

## Abstract

**Background and Purpose:**

Aiming to achieve light sedation via intranasal administration, this study showed that propofol (PPF) did not permeate across the rabbit nasal mucosa ex vivo from its marketed emulsion for injection.

**Experimental approach:**

Dilution of the emulsion with methyl-β-cyclodextrin in saline solution increased propofol solubility in water and diffusion across the nasal epithelium.

**Key results and conclusion:**

Despite these positive effects of the cyclodextrin, the amount of PPF permeated was minimal in 3 h, exceeding the formulation residence time in the nose. These results highlight the key role of formulation and the need for innovation in solubility and transmucosal transport enhancement techniques to optimize drug delivery and therapeutic efficacy.

## Introduction

Propofol (PPF) is an intravenous anesthetic and sedative drug extensively used in clinical practice. It is administered intravenously (IV) as a bolus injection or infusion [1]. It also exhibits dose-dependent amnestic and anxiolytic effects [2].

Procedural sedation and analgesia are of utmost importance in emergency medicine, both in the pre-hospital and hospital settings [3]. Despite pain being one of the primary reasons for access to the emergency department (ED) [4], the control of a patient’s anxiety and consciousness is a fundamental skill of the emergency physician [5], at any stage of life [6]. Sedation eases diagnostic and therapeutic procedures (*e.g*., luxation reduction, wound exploration, *etc.*), especially in poorly cooperative patients.

Multiple strategies and medications are available for pain control, some delivered via non-conventional drug administration routes like the nasal one. In contrast, fewer options exist for light sedation. In clinical practice, the ideal sedative should: 1) be rapidly administered; 2) show rapid onset and offset of action, possibly with a linear dose-response relationship; and 3) cause minimal and predictable side effects. PPF possesses all these properties and thus is widely used for anesthesia during surgery. Its use by non-anesthesiologists has been extended for sedating non-surgical patients [7,8].

PPF has very low solubility in water, as reported by Kazi *et al*. [9] and Date and Nagarsenken [10] without specifying the temperature (0.124 or 0.154 mg/ml, respectively). For this reason, it is formulated as a lipid-based liquid for injection. PPF raw material itself is a yellow oily liquid [11]. The marketed products are oil-in-water emulsions at 1 or 2 % PPF concentration (*e.g*., Diprivan^®^ by Aspen Pharma or the generic Propofol Kabi in Italy). Their IV bolus causes injection-site pain [12], whose origin and mechanism remain unclear. The drug itself, the lipid excipient, and the venous access are factors possibly involved in the pain experience.

Considering this and other drawbacks of IV PPF [2], a new concept could be to change the administration route, especially when light sedation, instead of deep anesthesia, is preferred. The intranasal (IN) route is intriguing for this purpose. In fact, several drug substances elicit a systemic effect upon delivery and deposition of the formulation inside the nasal cavities [13,14]. Midazolam and fentanyl are successfully employed for IN analgesia and sedation, especially in children [15,16]. Ketamine and dexmedetomidine, even in association, have been considered as well [17,18]. Following IN administration, anxiolytic and sedative effects may result from the double contribution of *i*) rapid absorption of the drug across the nasal epithelium and entry into the blood circulation and *ii*) direct nose-to-brain transport via the olfactory and trigeminal pathways [19]. The approach is non-invasive and relevant for uncooperative patients. It may also work for other sedative and anxiolytic agents in the ED, including propofol. Only one study investigated the sedation effect elicited by PPF emulsion administered intranasally to rats [20]. These authors found a pharmacodynamic response to IN Diprivan^®^ not significantly different from that of IN saline (negative control), whereas sedation was obtained with IN propofol-loaded chitosan amphiphilic nanoparticles. The greater response to the non-lipid nanoparticles *vs.* the lipid emulsion was attributed to an effect of the formulation.

Here, we assessed the transport of PPF across the rabbit nasal mucosa *ex vivo* because the hoped-for light sedation depends on the amount of drug released from the formulation and reaching the blood from the nasal cavity. The experiments were carried out by using Franz-type diffusion cells and rabbit nasal mucosa as model tissue [21]. The effect of methyl-β-cyclodextrin on PPF solubility and permeation was also evaluated [2].

## Experimental

### Materials

Propofol (PPF; 0.96 g/ml density at 25 °C) raw material (batch # FCB050002) was purchased from Fluorochem (Hadfield, UK) and used as an analytical standard for HPLC analysis. The commercial Propofol Kabi injection (PPF-FK; Fresenius Kabi, Isola della Scala, VR, Italy) was used for the permeation experiments as such or after dilution. PPF-FK is a milky white O/W emulsion for injection containing 10 mg/ml propofol. It is packaged in glass ampoules containing 20 ml of emulsion and was stored in the lab according to the manufacturer’s instructions (≤25 °C). Methyl-β-cyclodextrin (Me-β-CD; CAS 128446-36-6) was supplied by Sigma-Aldrich (Darmstadt, Germany). HPLC-grade acetonitrile and methanol were purchased by Merck KGaA (Darmstadt, Germany). All salts, reagents and other solvents were analytical grade.

### Methods

#### PPF equilibrium solubility study

The equilibrium solubility of PPF raw material was investigated in physiological saline solution (0.9 % w/v NaCl; from now on referred to as “saline”) using the liquid PPF raw material. An excess volume of PPF (about 200 μl) was added to 2 ml of the saline containing increasing concentrations of Me-β-CD, namely 0.07, 1.47 and 2.94 % (w/v). A fourth sample contained the saline without Me-β-CD. In all cases, liquid PPF was layered on top of the aqueous phase. Then, the vials were screw-capped and placed on an orbital shaker (100 rpm; 25 °C) for 22 hours. After 2 h of vial rest without shaking, a sample of the aqueous layer at the bottom was carefully withdrawn using a Hamilton syringe and centrifuged (10,000 rpm, 15 min). The collected samples were diluted with methanol for HPLC analysis. The experiment was conducted in duplicate.

#### PPF permeation experiments ex vivo

The *ex vivo* permeation experiments were carried out according to a consolidated protocol of the lab [22-25]. Vertical Franz-type diffusion cells (area 0.58 cm^2^) and excised rabbit nasal mucosa from slaughterhouse waste (Pola srl, Finale Emilia, MO, Italy) were used. The tissue was extracted within 2-3 h from the animal’s death and inserted between the cell donor and receptor compartments. After clamping the compartments together, the assembly tightness was verified by loading 200 μl of saline solution into the donor and checking for any leakage. Then, the lower compartment was accurately filled with 4.7 ml of Me-β-CD (0.07 % w/v) in saline as the receiving medium. After 15 min equilibration at 37 °C, the donor compartment was loaded either with 1 ml of PPF-FK emulsion as such (10 mg PPF loaded) or PPF-FK diluted 1:5 with saline without or with Me-β-CD (2 mg PPF loaded; 2.94 % w/v Me-β-CD).

The experiments lasted 3 hours with the medium magnetically stirred (250 rpm). At pre-determined time points, 0.5 ml of medium was sampled and replaced with an equal volume of fresh medium. After the last sampling, 100 μl of the residual formulation in the donor compartment was collected for PPF quantification and mass balance calculation. This aliquot was diluted with methanol, the obtained solution centrifuged (10,000 rpm, 5 min), and the supernatant injected in HPLC.

The mucosa specimen was collected, rinsed, and frozen. On the day of analysis, it was thawed and finely cut with a surgical blade to extract PPF with water and methanol by manual homogenization with a pestle. The resulting suspension was centrifuged (10,000 rpm, 10 min), and the supernatant was injected to quantify the PPF extracted. Finally, the samples withdrawn from the receptor were centrifuged (10,000 rpm, 10 min), and the supernatant was injected.

#### HPLC-UV method for propofol quantification

PPF concentration in the samples was determined by HPLC-UV on an Agilent Series 1100 apparatus with UV-Vis detection (software Chemstation v. B.04.03). The stationary phase was a Luna C18 column (3 μm, 150×4.60 mm; Phenomenex, Torrance, CA, USA). The mobile phase was composed of 20 mM KH_2_PO_4_ : acetonitrile (16:84), pH 3.0, for isocratic elution at 0.8 ml/min flow rate (backpressure 78-80 bar at *T* ≤25 °C). Absorbance was measured at 220 nm. In these conditions, the retention time of propofol was 3.2 minutes. The propofol standard solution was prepared by diluting 50 μl of PPF raw material into 20 ml with methanol (Stock solution). 50 μl of stock solution was further diluted to 10 ml with methanol (Standard solution; 12 μg/ml). Stock and standard solutions were freshly prepared on each day of analysis.

The method linearity was verified by analyzing a series of PPF solutions in methanol (1.0, 2.5, 5.0, 10.0, 20.0 μg/ml). Each concentration was injected in triplicate (*V*_inj_: 10 μl). A straight line was obtained in the considered concentration range (*y* = 28.951*x* + 0.9417; *R*^2^ = 0.9998). Moreover, using the signal-to-noise ratio method, the limit of quantification (LOQ) was determined at 0.25 μg/ml.

## Results and discussion

### Propofol solubility in saline was increased by methyl-β-cyclodextrin

In the 1990s Trapani and co-workers [26] first proposed the use of β-cyclodextrins to increase the solubility of PPF in water. Hydroxypropyl-β-CD (HP-β-CD) concentrations in the range of 4.05-40.5 % (w/v) increased PPF aqueous solubility up to 250 folds [26]. The cyclodextrin hollow truncated cone structure accommodates lipophilic molecules inside the hydrophobic cavity. The external surface is hydrophilic and provides solubility of the complex in water. Later, the solubilizing action of sulfobutylether 7-β-cyclodextrin (SBE-CD) was exploited, yielding, after compounding and lyophilization, a product with PPF concentration of 10 mg/ml [27]. Very recently, the interaction of PPF with HP-β-CD has been described by molecular modelling [2]. All this research shared the goal of increasing propofol solubility in water to develop aqueous-based injectable formulations, possibly reducing the cited drawbacks of lipid-based formulations.

HP-β-CD and SBE-CD are the most extensively used CD derivatives in marketed products for all administration routes except nasal [28]. As this study focused on PPF nasal administration, we selected methyl-β-CD (Me-β-CD) because it was used in an estradiol-containing marketed liquid nasal spray, currently discontinued (Aerodiol^®^, Laboratoires Servier, Suresnes, France). Moreover, the analgesic carfentanil complexed with dimethyl-β-cyclodextrin was absorbed more rapidly and extensively via the nasal route than intramuscular injection or oral administration [29]. About 2 to 5 % concentrations of Me-β-CD were reported not to damage the mucosal membrane, whereas irreversible cilia-inhibition was observed at 10 % Me-β-CD [30]. Me-β-CD works as a solubilizer and may directly act on cell membranes, ultimately enhancing the drug transmucosal permeability [31].

**Figure 1.**
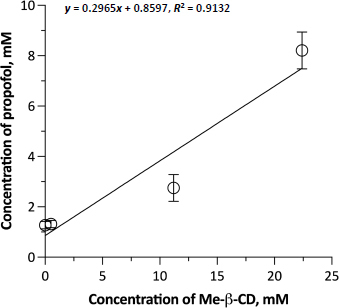
Phase solubility diagram of propofol in 0.9 % (w/v) NaCl saline solution as a function of methyl-β-cyclodextrin concentration at room temperature (≤25 °C). Data are reported as mean C SD (*n*=3).

Figure 1 shows the phase solubility diagram of PPF as a function of Me-β-CD concentration. The solvent of the Me-β-CD solutions was saline, not water, because of its suitable tonicity for the subsequent permeation experiments with the biological membrane. Me-β-CD increased the solubility of PPF in saline by about 5.5 folds across the considered concentration range (from 0.263 to 1.463 mg/ml). The Me-β-CD concentrations used were deliberately low and in a narrow range to remain below the 5% concentration limit to avoid mucosal damage. The lowest cyclodextrin concentration of the range was too small to exert an effect on PPF solubility. The fair linearity of the diagram (*R*^2^ = 0.9045) indicates that a 1:1 inclusion complex was formed. The calculated affinity *K*_1:1_ constant was 278.53 M^-1^. The inclusion complex was not isolated for further characterization. Nevertheless, Me-β-CD usefully improved the aqueous solubility of propofol at safe concentrations for nasal application.

### Propofol did not diffuse across the excised mucosa from the commercial emulsion

As mentioned, in their *in vivo* study, Uchegbu and co-workers [20] administered the injectable emulsion intranasally without seeing an effect. Thus, in the first *ex vivo* permeation experiment of the present work, the Propofol Kabi (PPF-FK) emulsion for injection was used as donor formulation (1 ml). It was found that PPF did not permeate the nasal tissue in 3 hours. It was never detected in the receptor compartment, and the mass balance confirmed that 100 % of the loaded PPF was still in the donor. We could exclude that the observed zero diffusion was due to low PPF solubility in the receptor medium; in fact, a preliminary transport experiment with Franz cells and a regenerated cellulose membrane (12-14 kDa MWCO) separating the compartments confirmed that sink conditions were respected (data not shown). This result could be explained by the lipophilic PPF remaining concentrated in the emulsion oil phase, with negligible partition toward the external aqueous phase for subsequent transmucosal diffusion. In this regard, by dialyzing 4 ml of PPF-FK in a regenerated cellulose dialysis tube (12-14 kDa MWCO) against 150 ml of an aqueous solution of 1 % hydroxypropyl-β-cyclodextrin [26] in saline, we verified that PPF was released very slowly. PPF concentrations out of the tube were 3.7 and 15 μg/ml after 3 and 24 hours, respectively. Several authors have determined the concentration of PPF in the aqueous phase of the emulsion (called “emulsion-free PPF”), mostly by dialysis methods over 24h, and reported similar values in the order of 10-17 μg/ml, corresponding to only 0.1% of the PPF concentration in the whole emulsion [32-34]. Hence, when the emulsion was deposited on the nasal membrane, its aqueous phase with such a very low PPF concentration mixed with mucus and led to an even lower PPF concentration in contact with the mucosa. In addition, the PPF partition did not favor the aqueous mucus, negatively affecting the concentration gradient across the mucosa and the diffusion. Yamakage *et al*. [33] also reported that the emulsion-free PPF decreased upon warming the emulsion from 20 to 36 °C. As the present permeation experiments were conducted at 37 °C, this temperature could have further reduced the concentration gradient for diffusion. In summary, the marketed injectable emulsion made PPF not (bio)available to cross the nasal mucosa.

### Emulsion dilution with methyl-β-cyclodextrin could contribute to propofol nasal permeation

Developing a new formulation for the intranasal administration of PPF was beyond the scope of this study. We chose to modify the commercial PPF-FK emulsion by increasing the volume of the external aqueous phase. This could facilitate the interaction of the emulsion with the mucus [35], which is described as an unstirred water layer (UWL) at the exterior surface of a lipophilic membrane, offering resistance to permeation, especially of poorly soluble drugs [36]. The emulsion was diluted 5 folds as instructed in the summary of product characteristics. The diluents were either saline solution alone or saline containing 2.94 % (w/v) Me-β-CD, *i.e.*, the maximum cyclodextrin concentration considered in the phase solubility study. Prior to the experiment, PPF was quantified in the aqueous phase of both dilutions after separating the oil and water phases by centrifugation (10,000 rpm, 20 min). PPF concentrations of 54.9 ± 0.8 μg/ml and 389.7 ± 3.4 μg/ml were measured, respectively without and with Me-β-CD. Thus, the dilution with saline slightly increased the “emulsion-free” PPF compared to the PPF concentration determined by dialyzing the commercial emulsion. More importantly, in the dilution made with the Me-β-CD-containing saline, the complexation of propofol by the oligosaccharide enabled more drug to partition from the oil to the water phase. It should be noted that both the above PPF concentrations were lower than the solubility values determined at 0 and 2.94 % (w/v) Me-β-CD concentrations in saline using PPF raw material (Figure 1). This difference was likely caused by the presence of the oil phase in the diluted emulsion, for which propofol maintained the greatest affinity in competition with cyclodextrin.

**Figure 2.**
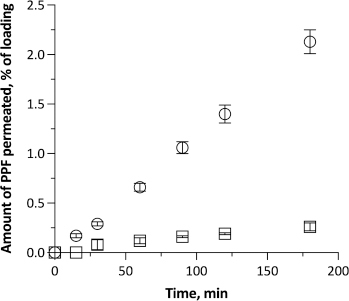
Propofol transport across excised rabbit nasal mucosa from 1:5 dilutions of the PPF-FK emulsion with: 0.9 % NaCl saline solution (square) and saline solution containing 2.94 % methyl-β-cyclodextrin (circle). Data are reported as mean c SEM (*n*=3).

Figure 2 shows the permeation of PPF from the two dilutions across excised rabbit nasal mucosa. The dilution without cyclodextrin led to a very small amount of PPF permeated in 3h (8.9 ± 1.3 μg cm^-2^), *i.e.*, less than 0.3 % of the PPF initially loaded in the donor. 0.4 % of PPF was extracted from the mucosa, and the rest was recovered from the donor (mass balance 97 %). The permeation was slow, with a 30-minute time lag, and quite variable. Still, some drug had diffused compared to the commercial emulsion, despite the dilution causing a 5-fold decrease of the total PPF concentration in the system. As measured, the dilution with saline had minimally increased the emulsion-free PPF concentration, which is the actual fraction of the drug available for diffusion at first.

When the PPF-FK emulsion was diluted 5 folds with the 2.94 % (w/v) Me-β-CD solution in saline, PPF permeation across the nasal membrane increased significantly. The time lag disappeared, signifying that some drug molecules were immediately ready to cross the tissue and diffuse. The linearity of the profile witnessed the early establishment of steady-state conditions maintained till the end of the test. The resulting steady-state flux was 0.41 ± 0.03 μg cm^-2^ min^-1^. 2.1 % of the loaded PPF diffused into the receptor compartment in 3 h, corresponding to 73.4 ± 4.1 μg cm^-2^ of PPF. On the one hand, this result was reasonably driven by PPF complexation by Me-β-CD. Despite the complexation that may determine a low concentration of free unbound drug in solution, a greater amount of drug dispersed at the molecular level, either bound or unbound, justifies the higher solubility in the aqueous phase. As discussed, this solubilizing effect by cyclodextrin shifted the PPF partition equilibrium between the oil and aqueous phases of the diluted emulsion, raising the emulsion-free drug concentration from 55 to 390 μg ml^-1^. Such a 7-fold higher concentration in the immediate vicinity of the biomembrane can justify the observed permeation. It enhanced the availability of drug molecules for permeation in the UWL. The drug molecules bound within the complex are in dynamic equilibrium with the unbound ones. While neither the bound molecules nor the complex itself permeate the membrane, unbound molecules do so. As a consequence of this flux, other bound molecules are released from the complex owing to the said equilibrium, thereby further sustaining the permeation process [36].

On the other hand, a permeation enhancement effect mediated by Me-β-CD is not excluded as the amount of PPF permeated was 9-fold greater than without cyclodextrin. Me-β-CD also led to more PFF accumulated within the membrane (1.2%). It is reported that cyclodextrins can form complexes with lipid components of the cell membranes [37]. This alters the integrity of the nasal barrier and may influence the elasticity of the nasal mucosa, ultimately leading to increased tissue permeability.

## Conclusions

A positive effect of Me-β-CD was seen on PPF diffusion across the rabbit nasal tissue, mainly due to a solubilizing action. Still, the permeation of the drug was minimal. The amount transported does not seem capable of inducing sedation because it was measured during 3 hours, much longer than the average residence time of a liquid inside the nose. PPF appears to be a difficult candidate for nasal administration, and a formulation study is recommended to improve its water solubility and transmucosal transport to reach blood levels that are useful for sedation. Liquid lipid-based formulations easily accommodate oily PPF, but do not release it enough in the intranasal aqueous environment. One exciting opportunity may be represented by nasal powders by exploiting the amorphous solid dispersion technology [38], similar to what has been done for other drugs candidates for nasal administration, *e.g.*, budesonide, which is even less soluble in water than PPF [39].
